# Long-term effect of stereotactic body radiation therapy for primary hepatocellular carcinoma ineligible for local ablation therapy or surgical resection. Stereotactic radiotherapy for liver cancer

**DOI:** 10.1186/1471-2407-10-475

**Published:** 2010-09-03

**Authors:** Jung Hyun Kwon, Si Hyun Bae, Ji Yoon Kim, Byung Ock Choi, Hong Seok Jang, Jeong Won Jang, Jong Young Choi, Seung Kew Yoon, Kyu Won Chung

**Affiliations:** 1 Department of Internal Medicine, College of Medicine, The Catholic University of Korea, Seoul, Korea; 2 Department of Radiation Oncology, College of Medicine, The Catholic University of Korea, Seoul, Korea

## Abstract

**Background:**

We evaluated the long-term effect of stereotactic body radiation therapy (SBRT) for primary small hepatocellular carcinoma (HCC) ineligible for local therapy or surgery.

**Methods:**

Forty-two HCC patients with tumors ≤ 100 cc and ineligible for local ablation therapy or surgical resection were treated with SBRT: 30-39 Gy with a prescription isodose range of 70-85% (median 80%) was delivered daily in three fractions. Median tumor volume was 15.4 cc (3.0-81.8) and median follow-up duration 28.7 months (8.4-49.1).

**Results:**

Complete response (CR) for the in-field lesion was initially achieved in 59.6% and partial response (PR) in 26.2% of patients. Hepatic out-of-field progression occurred in 18 patients (42.9%) and distant metastasis developed in 12 (28.6%) patients. Overall in-field CR and overall CR were achieved in 59.6% and 33.3%, respectively. Overall 1-year and 3-year survival rates were 92.9% and 58.6%, respectively. In-field progression-free survival at 1 and 3 years was 72.0% and 67.5%, respectively. Patients with smaller tumor had better in-field progression-free survival and overall survival rates (<32 cc vs. ≥32 cc, *P *< 0.05). No major toxicity was encountered but one patient died with extrahepatic metastasis and radiation-induced hepatic failure.

**Conclusions:**

SBRT is a promising noninvasive-treatment for small HCC that is ineligible for local treatment or surgical resection.

## Background

Hepatocellular carcinoma (HCC) is one of the most common malignant diseases [1-3]. Of the current therapeutic approaches for HCC, surgical resection and liver transplantation are used with curative intent for patients with small HCC [4]. However, the majority of HCC patients are ineligible for surgery because most have a cirrhotic liver with poor liver function or tumors are located in the central segments. Several modalities including radiofrequency ablation (RFA) and percutaneous ethanol injection (PEI) have been used to treat small HCC, but the optimal treatment remains controversial particularly for cases of small HCC ineligible for curative therapy.

Cyberknife (Accuracy Inc., Sunnyvale, CA) is a new stereotactic body radiation therapy (SBRT) that delivers a high dose of radiation in a short time to well-defined tumor sites. Cyberknife has been extended to extracranial SBRT applications [5-7] since it was invented for the treatment of intracranial lesions [8]. However, to date few studies have reported SBRT including Cyberknife for the treatment of intrahepatic tumors, especially primary HCC [9-14]. In our previous report [12], the overall response rate of non-resectable small HCC and portal vein tumor thrombosis (PVTT) in advanced HCC was 71.9% for a median follow-up of 10.5 months. This report expands our experience further, and discusses the long-term effect in primary HCC of Cyberknife SBRT that was limited to targeting parenchymal lesions ineligible for local ablation therapy or surgical resection.

## Methods

### Patient selection

**Figure 1:**
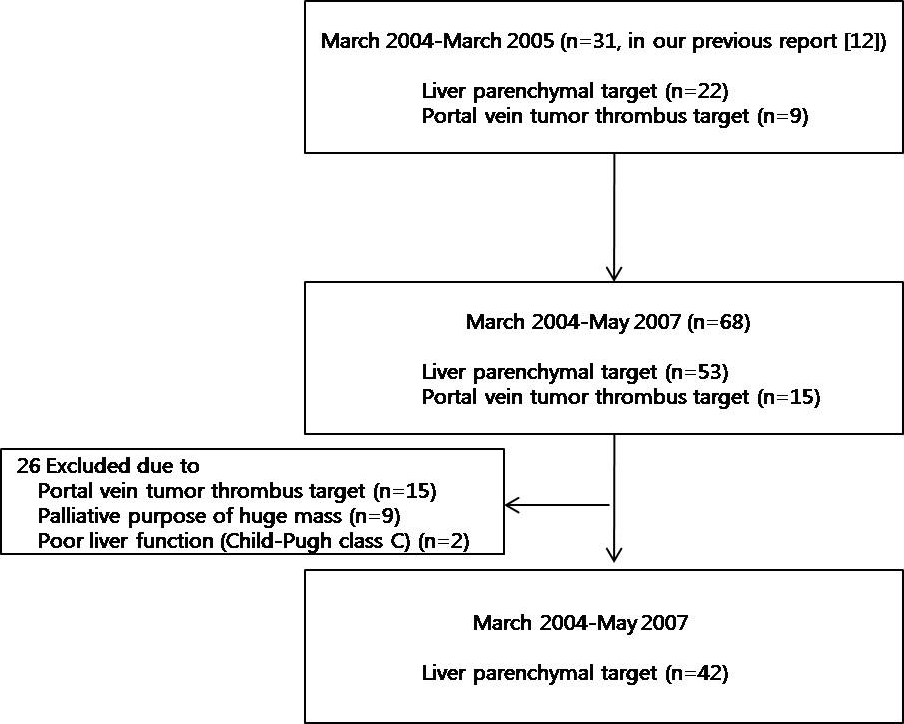
**Flow of study participants in the study **.

Sixty-eight HCC patients who underwent Cyberknife SBRT between March 2004 and May 2007 were initially considered. The study inclusion criteria for the tumor were primary HCC targeting parenchymal lesions without extrahepatic metastasis and a tumor volume of ≤ 100 cc [9], the presence of technical difficulties with local therapies such as RFA and PEI, an inoperable state because of poor liver function or inaccessible site, patient refusal to undergo surgery, or a viable remnant portion after previous treatment. All the patients were judged inoperable or inaccessible by a team consisting of a hepatobiliary surgeon, radiologist and hepatologist. Patient criteria were age ≤ 80, an Eastern Co-operative Group (ECOG) performance score of ≤ 2, preserved liver function (Child-Pugh class A and B), and no prior history of radiotherapy. According to the inclusion criteria, 15 patients with targeting the portal vein tumor thrombosis, nine patients treated for a huge HCC mass with palliative intent, and two patients with poor liver function (Child-Pugh class C) were excluded from the analysis. Thus, 42 HCC patients constituted the study cohort (Figure 1). HCC was diagnosed histologically or based on an elevated serum alpha-fetoprotein (AFP) level (> 400 ng/ml) with typical radiologic findings. Median follow-up after Cyberknife treatment was 28.7 months (range, 8.4-49.1) and median target tumor volume was 15.4 cc (range, 3.04-81.82). Written informed consent was obtained from all patients before treatment and this study was approved by the review board of our institution.

### Stereotactic body radiation therapy

SBRT was performed using a Cyberknife (Accuracy Inc.) image-guided radiosurgery system. To obtain radiographic landmarks, we percutaneously implanted four 3 × 1 mm gold seeds under ultrasonographic guidance in liver parenchyma near the tumor targets. On the following day, patients were vacuum immobilized in the supine position, and computed tomographic (CT) images were taken in spiral mode with a 2 mm slice thickness at maximum expiration. We used breath-holding techniques during Cyberknife treatment as described in our previous protocol [12].

**Figure 2:**
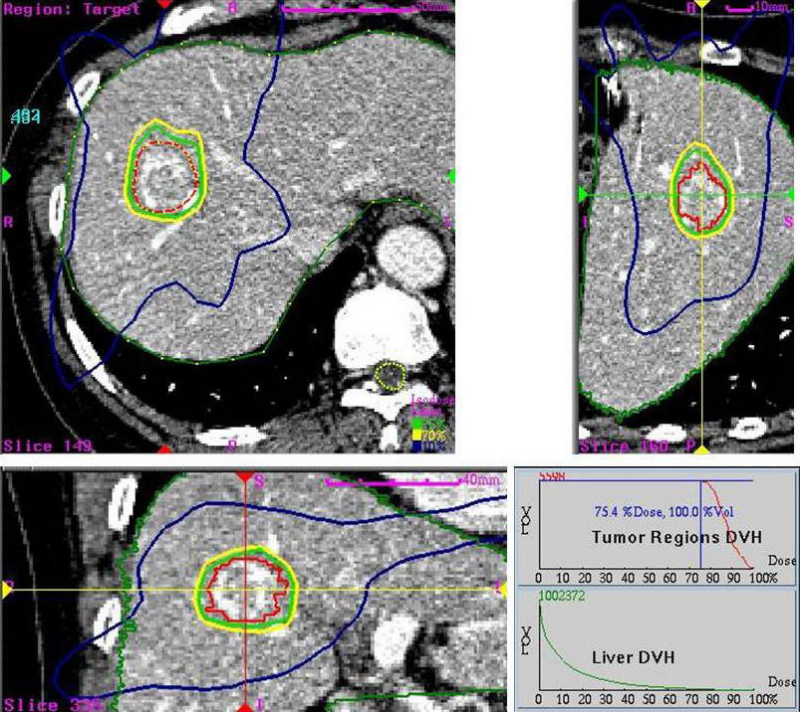
**GTV and isodose lines **. The dotted red line represents GTV, and the green, yellow, and purple curves the 75, 70 and 10% isodose lines, respectively. 36 Gy was prescribed on the 75% isodose line. Axial, coronal and sagittal views and dose-volume histogram (DVH) of the tumor and liver are demonstrated.

Gross tumor volume (GTV) was defined as contrast enhanced tumor volume on CT scans. Clinical tumor volume (CTV) was defined as a 2 mm margin around the GTV [15-17] and the planning target volume (PTV) was defined as a 3 mm margin around the CTV. In general, the superior-to-inferior movement was greater than in the left-to-right and anteroposterior directions. However, because we could not use 4-dimensional CT scans and there might be unexpected directional movement such as torsion or twisting of the tumor, our protocol defined the same margin as the CTV. The radiation dose was prescribed at the PTV with an isodose range of 70-85% (median 80%). The median total dose administered was 33 Gy (range, 30-39) delivered in three fractions on consecutive days. We decided the total dose mainly based on tumor size and tumor site, considering dose limitation to nearby normal tissues. We generally administered 30-36 Gy into the tumor which the volume was below 30 cc, however if the tumor site was distant from nearby organs, we administered 39 Gy even for a small HCC. Conformal shape inverse planning in the Cyberknife SBRT on-target treatment planning system (version 3.3) was used in the study (Figure 2).

### Dose limitation to normal tissues

The liver, esophagus, stomach, duodenum, intestine, kidneys, and spinal cord were contoured during the planning process and dose-volume histograms (DVH) were used to ensure that normal tissue tolerances were not exceeded. We limited the dose to normal tissues as in our previous protocol [12].

### Liver

We evaluated V20 as a predictor for liver damage accrued from the SBRT in our study: in the α/β ratio of 3, 30-35 Gy with conventional fractionation is equivalent to a dose of 3 × 6 Gy (total, 18 Gy) to the whole liver. The V20 was limited so as not to exceed 50% of the functional whole liver tissue.

### Stomach, duodenum and intestine

Because of the lack of clinical data on the effect of very high fractional doses exceeding 8 Gy, a dose of 7 Gy was chosen based on the experience in brachytherapy. Therefore, the maximum dose to the stomach, duodenum and intestine was limited to below 7 Gy per fraction (total, 21 Gy).

### Kidney

In this study, at least two-thirds of the right kidney was limited to receiving a dose of less than 5 Gy per fraction (total, 15 Gy). With an α/β ratio of 3, 23 Gy with conventional fractionation is equivalent to a dose of 3 × 5 Gy (total 15 Gy).

### Spinal cord

The maximum dose to the spinal cord was limited to below 7 Gy per fraction from the linear-quadratic formula of Withers et al. For an α/β ratio of 3, 42 Gy with conventional fractionation is equivalent to a dose of 3 × 7 Gy (total, 21 Gy)

### Assessment of response and toxicity

Patients underwent dynamic CT scans 1 month after completion of SBRT, and then tumor response was checked at 2-3 month intervals. Treatment response and local recurrence were evaluated using follow-up dynamic CT scans and serum alpha-fetoprotein (AFP). MRI scans and/or Positron emission tomography (PET) CT scans were used to discriminate the vague lesion or response and evaluate overall response in some cases. In patients who experienced local recurrence or intrahepatic metastasis after Cyberknife treatment, transarterial chemoembolization (TACE), local ablation therapies, or liver transplantation were recommended.

In-field and overall tumor responses were defined principally according to the amended Response Evaluation Criteria in Solid Tumors (RECIST) criteria [18], taking into account tumor necrosis recognized by non-enhanced areas. An in-field response was defined as target lesion response within the irradiated field. Complete response (CR) was defined as the disappearance of any intratumoral arterial enhancement in all target lesions. Partial response (PR) was defined as at least a 30% decrease in the sum of the diameters of viable (contrast enhancement in the arterial phase) target lesions, taking as a reference the baseline sum of the diameters of target lesions. Progressive disease (PD) was defined as at least 20% increase in the sum of the diameters of viable (enhancing) target lesions or the appearance of a new lesion, and stable disease (SD) was defined as a tumor status that did not meet the above three response criteria. Initial in-field response was evaluated by determining the maximum reduction rate within 12 months following Cyberknife treatment. In-field progression was defined as any progression of the target lesion within the irradiated field after initial response to Cyberknife treatment. In-field progression-free survival was calculated from the date of initial response to the date of in-field progression. Overall in-field response was defined as target lesion response at the end of follow-up or the date of death. Overall response was defined as the whole body response considering hepatic out-of-field progression and extrahepatic metastasis in addition to the hepatic in-field response at the end of follow-up or the date of death.

Serum AFP response was also evaluated in patients with a serum AFP level greater than normal before Cyberknife treatment. Based on percentage changes versus baseline levels, AFP responses were categorized as CR (AFP normalization), PR (an AFP reduction of ≥ 50%), PD (an AFP increase of ≥ 25%), and SD (an AFP change that did not meet the above three response criteria) [19].

Toxicities were assessed using the Radiation Therapy Oncology Group (RTOG) guidelines [20,21]. Acute toxicity was assessed weekly until 90 days post-treatment. Late toxicity was defined as a toxicity occurring at > 90 days post treatment. For both acute and late toxicities, grade 3-4 indicates major toxicity.

### Statistical analysis

Data are expressed as mean ± SDs, medians (ranges), or rate. Statistical analyses were performed using SPSS version 14.0 for Windows (SPSS Inc., Chicago, IL). Patient survival was calculated from the date of Cyberknife treatment until the date of death or last follow-up. Cumulative survival rates were estimated using the Kaplan-Meier method. The factors affecting the survival rate were identified on univariate and multivariate analysis using Cox's proportional hazard model.

## Results

### Patient characteristics

Patient baseline characteristics are listed in Table 1. The patients included 32 men and 10 women aged 60.1 ± 10.9 years. Hepatitis B virus infection was the most common cause of HCC (69.0%). Ninety percent of patients were Child-Pugh grade A, and all patients had an ECOG performance grade of 0 or 1. Fifteen (35.7%) patients had multifocal tumors, but the target lesion in these patients was a single viable remnant lesion among necrotic tumor masses. Eight (19.0%) patients were treatment naïve, and the others had been treated with TACE alone or with combined local therapies before Cyberknife treatment.

**Table 1:** Basal characteristics of patients

Patient Characteristics	N = 42
Overall median follow up period (months, range)	28.7 (8.4-49.1)
Age (years, mean ± SD)	60.1 ± 10.9
Sex (male/female)	32/10
Cause of HCC (HBV/HCV/Alcohol/Others)	29/7/2/4
ECOG performance (0/1)	41/1
Child-Pugh classification (A/B/C)	38/4/0
AJCC stage (I/II/IIIa/IV)	23/16/3/0
Median tumor volume (cc, range)	15.4 (3.04-81.82)
Tumor number (solitary/multifocal)	27/15
Initial median AFP (ng/dl, range)	13.9 (1.0-798.8)
Previous therapy	
No treatment	8
TACE alone	16
TACE combined with locoregional therapies	16 (PEI 13, RFA 3)
TACE combined with surgical resection	2

Abbreviations: HCC, hepatocellular carcinoma; HBV, hepatitis B virus; HCV, hepatitis C virus; ECOG performance, Eastern Co-operative Group performance; AFP, alfa-fetoprotein; TACE, transarterial chemoembolization; PEI, percutaneous ethanol injection; RFA, radiofrequency ablation

### Tumor and AFP response

**Figure 3:**
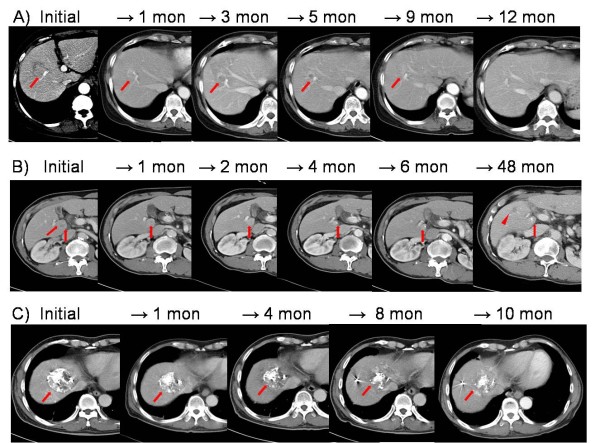
**In-field tumor response after Cyberknife treatment **. A) A case of complete response. B) A case of partial response. C) A case of stable disease. (Arrow: target lesion, arrowhead: intrahepatic metastasis)

**Figure 4:**
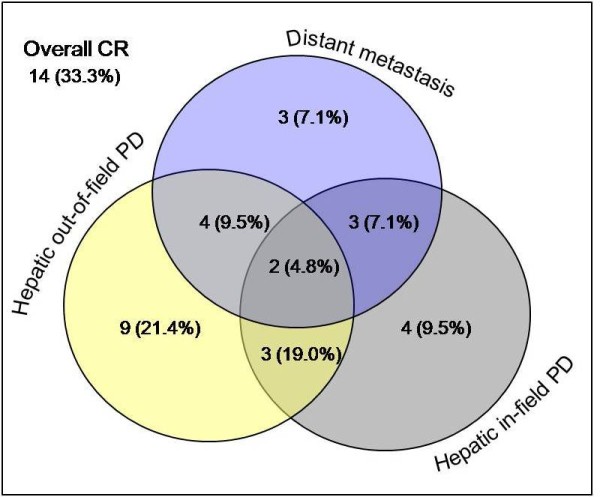
**Pattern of disease recurrence **. CR; complete response, PD; progressive disease

Initial in-field responses and overall in-field responses are shown in Table 2: CR for 25 patients (59.6%, Figure 3A); PR for 11 patients (26.2%, Figure 3B); and SD for 6 patients (14.3%, Figure 3C). No PD response was observed for an in-field lesion. The mean time to achieve CR or PR was 5.1 ± 3.7 months. During the follow-up period, in-field progression eventually occurred in 12 patients (28.6%); all the patients with SD and half of those with PR for the initial in-field lesions. Thus, all 25 patients with CR as an initial in-field response maintained in-field CR throughout follow-up. Apart from those with in-field CR, 11 patients without in-field progression developed hepatic out-of-field progression (n = 6), distant metastasis (n = 2) and both (n = 3) during the follow-up period. In terms of overall response during follow-up, 14 patients (33.3%) achieved CR and 28 (66.7%) patients had PD. Sixteen (57.1%) of the 28 PD patients showed no in-field progression after Cyberknife treatment but developed out-of-field progressions. Overall, hepatic out-of-field progression occurred in 18 patients (42.9%) and distant metastasis developed in 12 patients (28.6%)(Figure 4).

**Table 2:** Tumor and AFP response

Response	In-field response		Overall In-field response	Overall response	AFP response
				
	Initial response → Progression				
CR	25 (59.6)	0	25 (59.6)	14 (33.3)	10 (45.5)
PR	11 (26.2)	6 (54.5)	5 (11.9)	0	4 (18.2)
SD	6 (14.3)	6 (50.0)	0	0	4 (18.2)
PD	0	0	12 (28.6)	28 (66.7)	4 (18.2)
Total	42 (100)	12 (28.6)	42 (100)	42 (100)	22

Abbreviations: CR, complete response; PR, partial response; SD, stable disease; PD, progressive disease

After Cyberknife treatment, 25 patients (59.5%) who experienced local recurrence or intrahepatic metastasis underwent salvage therapy such as TACE and/or local therapy or liver transplantation.

Serum AFP response was assessed in the 22 evaluable patients with an increased AFP level at baseline (Table 2). Overall, 10 (45.5%) and four (18.2%) of these patients achieved CR or PR for AFP, respectively. In the follow-up period, 4 of the 22 patients achieving overall CR maintained CR for AFP. Of the 18 of the 22 patients having overall PD, seven showed CR, two PR, one SD and eight PD for AFP. The seven patients with overall PD who showed CR for AFP did not develop extrahepatic metastasis, but developed in-field or hepatic out-of-field progression. However, there was no significance between other patterns of final AFP response and tumor progression type.

**Figure 5:**
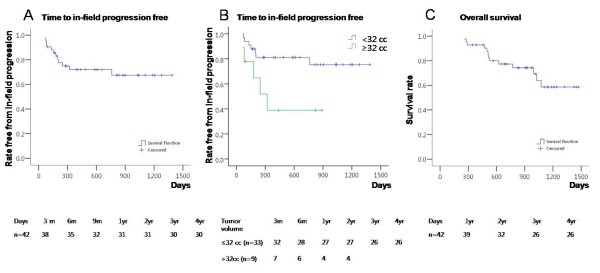
**In-field progression-free survival rates and overall survival rates **. A) In-field progression-free survival rates. B) Patients with a tumor volume of < 32 cc had longer in-field progression-free survival rates than those with a tumor volume of ≥ 32 cc (*P *= 0.026 by log rank test). C) Overall survival rates.

In-field progression-free survival rates at 6 months, 1 year and 3 years were 83.0%, 72.0% and 68.0%, respectively, with a median progression-free interval of 15.4 months (Figure 5A). In general, greater tumor volume was associated with more in-field progression. Patients with a tumor volume of < 32 cc had better in-field tumor responses and in-field progression-free survival rates than those with a tumor volume of ≥ 32 cc (*P *= 0.026, log rank test). The in-field progression free survival rate at 1 year with a tumor volume of < 32 cc was superior to that with a tumor volume of ≥ 32 cc (81.1% vs. 38.9%, Figure 5B). The area under the Receiver Operation Characteristic curve analysis of tumor volume for predicting in-field progression was 0.676 (*P *= 0.077) and a tumor volume of ≥ 32 cc showed a sensitivity of 45.5% and a specificity of 83.3%.

Overall survival rates at 1-year and 3-year after Cyberknife treatment were 92.9% and 58.6%, respectively (Figure 5C). When analyzing the factors affecting survival, initial in-field response, in-field progression, a tumor volume of < 32 cc and initial Child-Pugh score were significant factors (Table 3). In multivariate analysis, a tumor volume of < 32 cc and no distant metastasis were associated with survival.

**Table 3:** Factors identified on univariate and multivariate analysis as influencing the survival

	Univariate analysis	Multivariate analysis	
	
	P	Hazard ratio (95% Confidence Interval)	P
Initial In-field response	0.003		
In-field progression	0.006		
Hepatic out-field recurrence	0.106		
Distant metastasis	0.167	15.495 (1.298-184.896)	0.030
Tumor stage	0.218		
Initial tumor volume 32 cc (< vs. ≥)	0.005	6.328 (1.126-35.574)	0.036
Child-Pugh classification score	0.023		
Age	0.822		

### Adverse events related to treatment

The most common acute events were constitutional symptoms (34%), elevated liver enzyme (30%), and leucopenia (18%), but all improved without requiring specific management (grade 1 or 2)(Table 4). In one patient there was a targeting error because the true lesion located in the hepatic angle was moved by spontaneous large bowel movement. This patient was retreated 1 week after initial Cyberknife treatment. The missed target lesion showed necrosis on CT scan after 1 month, but no complications developed. One male patient experienced a major late toxicity (grade 4). This patient showed progressive elevation of total bilirubin and alkaline phosphatase without liver enzyme elevation combined with cancer recurrence and bone metastasis and died from hepatic failure 20 months after Cyberknife treatment. He had a tumor volume of 35.1 cc for Cyberknife treatment, and 30 Gy had been prescribed.

**Table 4:** Adverse events related to treatment

Toxicity	Acute (%)		Late (%)
**grade**	**Grade 1**	**Grade 2**	**Grade 4**

Constitutional symptoms	15	0	0
Leukopenia	5	3	0
Elevated liver enzyme	5	8	0
Elevated bilirubin and alkaline phosphatase	0	1	0
Liver failure	0	0	1
Other (target error)	0	1	0

## Discussion

The results of this study show excellent in-field responses to Cyberknife SBRT in HCC within tumors < 100 cc in volume that are ineligible for local ablation therapies or surgical resection. Furthermore, all patients that initially achieved CR for in-field lesions experienced no in-field progression during the follow-up period.

Surgical resection is best indicated for patients with a single tumor and well-preserved liver function, and such patients may achieve a 5-year survival rate of 60-70% [22,23]. In the present study, the 3-year survival rate for Cyberknife SBRT was 58.6%, which was slightly lower than for surgical resection. In view of the large proportion of multifocal tumors in our study population and the difficult tumor sites for operation, we find this survival rate acceptable [4,24]. Other local treatment methods, especially RFA, are emerging as alternative curative options for patients unsuitable for surgery or liver transplantation [4]. Complete tumor necrosis by RFA was pathologically shown by 83% of tumors < 3 cm [25], but proximity of lesions to the gall bladder or main vessels, a sub-diaphragmatic location, or the presence of a non-echogenic lesion present major problems for the use of RFA [26]. Although radiotherapy alone or in combination with TACE has become a potential treatment option for advanced HCC, it is not considered as first-line treatment for small HCC [27,28]. Doses of 30-35 Gy with conventional fractionation are often considered to be the limit of liver tolerance. However, the role of radiotherapy for the treatment of small HCC or portal vein thrombosis has recently been emphasized in the context of the development of SBRT. This is because the radiation dose delivered by SBRT rapidly falls off at the periphery of target lesions, which enables the accurate delivery of high doses of radiation to a specified lesion with hypofractionation as opposed to traditional protracted treatment courses over several weeks. We did not intend to compare RFA/surgical resection and SBRT in small HCC, but we suggest that SBRT is an alternative option in case of ineligible for the former treatment.

We reviewed the reports of SBRT including more than 15 cases of its use for primary HCC (Table 5) [9,11]. Blomgren et al. first reported the use of hypofractionated SBRT for the treatment of extracranial malignancies [29]. Although several reports on hypofractionated SBRT applied to intrahepatic tumors, such as cholangiocarcinoma and metastatic tumors were subsequently published [9-11,15-17,30-32], little has been published on its performance in primary HCC. In the studies shown in table 5, median follow-up periods ranged from 17.6 to 28.7 months, median tumor volume ranged from 13.6 to 173 cc and local control rates were 65%-100% at 1-2 years [9,11]. Local failure was usually defined as recurrence of the target tumor, the demonstration of new enhancement, or RECIST progressive disease, although definitions of local control vary between the studies. Takeda et al. [9] reported the highest local control rate to date at > 90%, which is probably attributable to a tumor volume of < 100 cc and the fact that 14 of their 16 patients underwent combined TACE 2 weeks prior to SBRT. Furthermore, in that study [9] SD lesions might have been included in the local control group, because RECIST responses were not shown. Tse et al. [11] reported the lowest local control rate, 65% at 1 year, presumably, because tumor volumes were large (median 170 cc). Although Wulf et al. [16] reported a local control rate of 100% for a median tumor volume of 114 cc (range, 14-516), the overall survival rate was only 20% at 2 years because of intrahepatic metastasis and progression. In contrast, we did not achieve outstanding local control rates (in-field progression-free rate) at 1 and 2 years, but the overall survival rates at 1 and 3 years, 92.9% and 58.6%, were better. In addition, it was interesting to find that patients who achieved initial in-field CR maintained this status throughout follow-up, which suggests that initial in-field response and in-field progression are important overall survival indicators. However, we should note the 11 patients in the initial in-field CR developed hepatic out-of-field progression and distant metastasis without in-field progression. Because SBRT is a local treatment and a tumor volume of < 32 cc and distant metastasis were significant factors for survival, regular monitoring for target lesion and distant metastasis is essential.

**Table 5:** Hypofractionated radiotherapy trials on primary and metastatic hepatic tumors

Author	Lesions (HCC/CCC/Metastatic tumor)	Treatment (prescription dose/fractionated frequency/isodose)	Median follow-up months (range)	Local control rate *	Survival rate	Median tumor volume cc (range)	Toxicity
Takeda [9] 2008	16/0/0	35(20-50) gy/5-9/80%	20.4 (8.1-33.1)	100% at 1 year, 94% at 2 year	100% at 2 year	13.6 (3.4-72)	No SE

TSE [11] 2008	31/10/0	36 (24-54) gy/6/NA	17.6 (10.8-39.2)	65% at 1 year (CR 5%, PR 44%, SD 42%)	51% at 1 year (HCC 48% at 1 year)	173 (9-1913)	No SE 5 patients (12%); grade 3 (liver)

Present	42/0/0	33 (30-39) gy/3/80%	28.7 (8.4-49.1)	72.0, 67.5% at 1 and 2, 3 and 4 year (CR 60%, PR 26%, SD 14%)	92.9/77.3/58.6/58.6% at 1, 2, 3, 4 year	15.4 (3.0-81.8)	1 patient (2%); grade 4 (liver)

Abbreviations: HCC, hepatocellular carcinoma; CCC, cholangiocarcinoma; NA, non-available

*Local control rates are in-field progression free rates or tumor responses according to RECIST criteria.

The use of Cyberknife SBRT as a method for hypofractionated SBRT to treat primary HCC is an important aspect of our study. A phase I dose escalation trial of Cyberknife SBRT in liver tumors was published in abstract form in the American Society of Clinical Oncology (ASCO) in 2006 and 2007 [13,14] and our preliminary findings were reported in 2008 [12]. The 2006 ASCO report of 12 unresectable primary or metastatic liver tumors with a mean volume of 27.1 cc and a median follow-up of 6 months demonstrated an interval decrease in size for six lesions and stable disease for three lesions. According to the ASCO 2007 report, Cyberknife SBRT-treated primary liver or metastatic tumors achieved 24% CR and 40% PR over a median follow-up of 7 months. These outcomes are better than those mentioned in prior reports for other hypofractionated SBRT using the conventional LINAC. In our previous study [12], CR and PR for the small HCC group that was not the PVTT target group were 26.1% and 56.3%, respectively. Our study shows better responses (CR 59.6% and PR 25.5%) than the ASCO reports [13,14], although we analyzed only primary HCCs of ≤ 100 cc. The advantage of the present study is that Cyberknife SBRT was used to treat a large number of primary HCC and patients were followed over a relatively long follow-up period.

At the time we began the use of Cyberknife for HCC, there was no report identifying the safe dose. In terms of toxicity, the present study shows that Cyberknife SBRT is feasible and safe in primary HCC, which is consistent with the findings of a previous phase I study [13,14] and our previous study [12]. One of our patients died as a result of radiation-induced hepatic failure 20 months after Cyberknife treatment, but he also experienced combined tumor progression and metastasis. Takeda et al. and Tse et al. also reported no serious SBRT-related toxicities [9,11]. A targeting error suggests the importance of tumor location, particularly in the hepatic angle or near the beating heart, and of control breath holding. We contoured the liver, stomach, duodenum, intestine, kidney, and spinal cords during the planning process and used DVH to ensure that normal tissue tolerances were not exceeded. We plan to escalate the total dose 42-45 Gy in three fractionation in case of favorable tumor sites, taking into consideration the limitation dose to normal tissues.

## Conclusions

In the present study, excellent in-field responses were obtained for Cyberknife SBRT in HCC. Notably, all patients who initially achieved CR for in-field lesions maintained in-field CR during the follow-up period (median 28.7 months). Furthermore, this study shows that Cyberknife SBRT is a feasible, effective treatment for primary HCC, and suggests that Cyberknife SBRT be considered a promising noninvasive modality when small HCC of < 32 cc is inappropriate for surgical resection or ablation therapy. Further study is required to define the effects of administered radiation dose and fractionation, and to determine toxicities in selected patients with small HCC.

## Competing interests

This paper is original and it has not been published or accepted for publication, either in whole or in part, in any form. We did not have any financial support or relationships that could pose a conflict of interest.

## Authors' contributions

JHK, SHB, JYK, BOC, HSJ, JWJ, JYC, SKY, and KYC made substantial contributions to the conception and design of the study. JHK and SHB participated in the design of the study, performed the statistical analysis and interpretation of data, and drafted the manuscript. JYK, BOC, and HSJ carried out the Cyberknife treatment and participated in the analysis of tumor response. JWJ, JYC, SKY and KYC participated in study design and coordination and helped to draft the manuscript. All authors read and approved the final manuscript.

## Pre-publication history

The pre-publication history for this paper can be accessed here:

http://www.biomedcentral.com/1471-2407/10/475/prepub
